# Risk Factors Associated With the Case Fatality Rate of Pulmonary Tuberculosis in Pregnancy: A Five-Year Retrospective Study From a Developing Country

**DOI:** 10.7759/cureus.48446

**Published:** 2023-11-07

**Authors:** Ernawati Ernawati, Rizka E Prasetya, Aditiawarman Aditiawarman, Agus Sulistyono, Muhammad Ilham A Akbar

**Affiliations:** 1 Obstetrics and Gynaecology, Universitas Airlangga, Surabaya, IDN

**Keywords:** human immunodeficiency virus, infection, maternal mortality, miliary tuberculosis, pregnancy, pulmonary tuberculosis

## Abstract

Background

Tuberculosis is a leading cause of maternal and fetal mortality in women of reproductive age. Tuberculosis is frequently misdiagnosed and treated inadequately during pregnancy. Although the global case fatality rate of tuberculosis is decreasing annually, the trend of tuberculosis mortality in Indonesia remains relatively high. Most tuberculosis reports do not include pregnancy status because most countries do not routinely screen for tuberculosis in pregnant women and do not report pregnancy status in female cases. In Southeast Asia, there is currently insufficient data regarding the risk factors associated with maternal mortality due to tuberculosis. This study aimed to identify the risk factors associated with tuberculosis-related mortality during pregnancy.

Methodology

This retrospective study was conducted at Dr. Soetomo General Hospital, Surabaya. Data were collected from patients’ medical records. The samples were all pulmonary tuberculosis cases in pregnancy (suspected, bacteriological, and radiologically confirmed cases) from 2014 to 2018. Data on maternal characteristics, underlying risk factors, and maternal outcomes in pregnant women with tuberculosis were collected from medical records. A total of 77 cases of pulmonary tuberculosis in pregnancy were obtained and analyzed using the chi-square test for differences between pregnant women with tuberculosis who survived and those who did not.

Results

In total, 77 cases of pulmonary tuberculosis out of 7,242 deliveries were found during the past five years (incidence per year was 1.07), of whom 20.8% (16/77) died. Eight patients died before the gestational age reached 28 weeks. Most of the non-surviving women were aged <35 years (93.8%; 15/16). More than 30% (5/16) of the patients had human immunodeficiency virus co-infection, and the highest risk factors were pneumonia and miliary tuberculosis. Miliary tuberculosis was significantly associated with maternal mortality in pulmonary tuberculosis (p = 0.004) with a relative risk of 3.43.

Conclusions

According to the findings of this study, miliary tuberculosis is a significant risk factor for maternal mortality during pregnancy.

## Introduction

Tuberculosis is a leading cause of maternal and fetal mortality in women of reproductive age. According to the WHO Tuberculosis Report 2014, there were 3.3 million female tuberculosis cases in 2013, with 510,000 deaths, and one-third of these women had human immunodeficiency virus (HIV) co-infection. This report did not include pregnancy status because the majority of countries did not routinely screen for tuberculosis in pregnant women and did not report pregnancy status in female cases [1]. In high-burden countries, the prevalence of active tuberculosis in pregnancy and postpartum was greater than 60 cases per 100,000 people per year, whereas it was less than 20 cases per 100,000 people per year in low-burden countries [2]. The highest number of tuberculosis cases in 2016 (45%) occurred in Southeast Asia, where Indonesia was included and classified as a high-burden nation for tuberculosis. Although the global case fatality rate of tuberculosis decreases by 3% per year, the trend of tuberculosis mortality in Indonesia remains relatively high [3]. The incidence of tuberculosis in Indonesia was greater than the global average in 2016, with 1.02 million cases (391 cases per 100,000 population) and a mortality rate of 37 cases per 100,000 individuals. Among all the cases reported in Indonesia, 41.7% were female [4].

Although pregnancy does not affect tuberculosis pathogenesis, reactivation of latent infection, or therapy response, the symptoms of tuberculosis in pregnancy are similar to those in non-pregnant patients, including fever, cough, weight loss, night sweats, and malaise. As a result, tuberculosis is frequently misdiagnosed and inadequately treated during pregnancy [5]. Most mortality associated with tuberculosis is due to non-tuberculosis factors (kidney failure, malignancy, and cirrhosis), with miliary tuberculosis and pneumonia being the leading causes of mortality associated with tuberculosis [6]. The co-occurrence of tuberculosis and human immunodeficiency virus/acquired immunodeficiency syndrome (HIV/AIDS) has significantly amplified mortality rates among women during their reproductive years. Both disorders are considered risk factors that contribute to higher maternal mortality in Sub-Saharan Africa [7]. In Southeast Asia, there is currently insufficient data regarding the risk factors associated with maternal mortality due to tuberculosis. Therefore, this study aimed to identify risk factors associated with tuberculosis-related maternal mortality during pregnancy.

## Materials and methods

This retrospective study was conducted at Dr. Soetomo General Academic Hospital, the largest referral hospital in East Indonesia. The data were obtained from the patient’s medical records. The study sample consisted of pregnant women admitted to the hospital’s delivery room between 2014 and 2018. These women were suspected cases, bacteriologically proven cases, or radiologically confirmed cases of pulmonary tuberculosis. Their medical records were thoroughly documented and included in the analysis. This study employed a comprehensive sampling approach, wherein individuals with incomplete medical record data were eliminated from the analysis. Data on several maternal variables, including maternal age, gestational age, parity, HIV status, presence of intrauterine growth restriction (IUGR), and mode of delivery, were collected.

Additionally, information on underlying risk factors and maternal outcomes, including survival or non-survival, was gathered. SPSS version 23 (IBM Corp., Armonk, NY, USA) was employed for data analysis. The chi-square test was utilized to examine the distinct characteristics among the cohorts of pregnant women diagnosed with tuberculosis who survived and those who did not. Before starting the investigation, the research protocol underwent a comprehensive evaluation and received approval from the Ethics Committee of Dr. Soetomo General Academic Hospital under reference number 0119/LOE/301.4.2/IX/2020.

## Results

**Figure 1:**
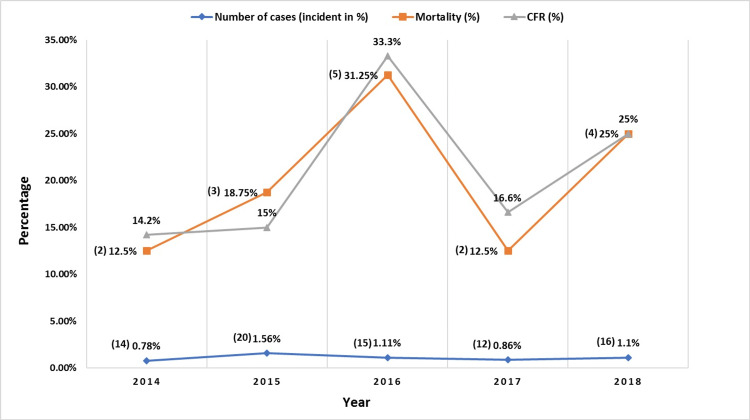
The number of cases, incidence, and case fatality rate of tuberculosis in pregnancy at Dr. Soetomo General Academic Hospital, Surabaya (2014-2018)

There were 77 cases of pulmonary tuberculosis out of 7,242 births over the course of five years, of whom 16. Figure 1 displays the data trends, case fatality rates, and case years. The annual incidence was 1.07%, and the average case fatality rate over the past five years was 20.82%.

The characteristics of pregnant women with pulmonary tuberculosis are shown in Table 1. Most births were performed via cesarean section. The most prevalent indications were obstetric reasons (abnormal fetal presentation and dystocia) and fetal distress, and there were 12 cases with indications of severe lung damage and deteriorating maternal conditions. In contrast, only 13 patients underwent vaginal delivery (three in the non-survival group). Eight patients in the survivor group were not delivered because of early gestation.

**Table 1:** Clinical characteristics of pregnant women with tuberculosis in Dr. Soetomo General Academic Hospital. IUGR = intrauterine growth restriction; HIV = human immunodeficiency virus

Clinical Characteristics	N (%)
Age (year)	
<35	64 (83.1)
≥35	13 (16.9)
Gestational age (weeks)	
<28	27 (35.1)
28–34	22 (28.6)
>34	28 (36.4)
Parity	
Primipara	21 (27.3)
Multipara	56 (72.7)
Maternal outcome	
Survived	61 (79.2)
Did not survive	16 (20.8)
HIV status	
Positive	14 (18.1)
Negative	50 (64.9)
Unknown	13 (16.8)
IUGR	6 (7.7)
Mode of delivery	
Vaginal birth	13 (19.5)
Cesarean section	40 (51.9)
Not delivered	24 (31.2)

Table 2 presents the factors associated with maternal mortality in pulmonary patients. Eight of the 16 pregnant women who died of pulmonary tuberculosis died before 28 weeks of gestation, and the majority of the non-survivors were under 35 years of age (15/16). There was a higher mortality rate among pregnant women aged 35 years owing to a variety of complications. Two patients had HIV co-infection with miliary tuberculosis, one patient had heart defects, one patient had HIV co-infection and pneumonia, six patients were co-infected with bacterial pneumonia, and three patients had miliary tuberculosis. More than 30% of individuals were HIV positive (5/16).

**Table 2:** Maternal factors associated with maternal mortality with pulmonary tuberculosis at Dr. Soetomo General Academic Hospital. *: chi-square test; **: including preeclampsia, eclampsia, and chronic hypertension. HIV = human immunodeficiency virus; PROM = premature rupture of membrane

Maternal characteristics	Survivor	Non-survivor	P-value*
	(N = 61)	(N = 16)	
	N (%)	N (%)	
Maternal age (years)			0.202
<35	49 (80.3)	15 (93.8)	
≥35	12 (19.7)	1 (6.3)	
Gestational age (weeks)			0.082
<28	19 (31.1)	8 (50)	
28–34	16 (26.2)	6 (37.5)	
>34	26 (42.6)	2 (12.5)	
Parity			0.819
Primipara	17 (27.9)	4 (25)	
Multipara	44 (72.1)	12 (75)	
Mode of delivery			0.122
Vaginal birth	10 (16.4)	3 (18.8)	
Cesarean section	35 (57.4)	5 (31.2)	
Not delivered	16 (26.2)	8 (50)	
HIV status			0.268
Positive	9 (14.8)	5 (31.3)	
Negative	42 (68.9)	8 (50)	
Unknown	10 (16.4)	3 (18.8)	
Risk factor			
Hypertensive disorder**	11 (18)	0 (0)	0.067
Heart disease	4 (6.6)	1 (6.3)	0.965
Pneumonia	15 (24.6)	7 (43.8)	0.131
PROM	3 (4.9)	2 (12.5)	0.273
Miliary tuberculosis	4 (6.6)	5 (31.3)	0.004

Pneumonia and miliary tuberculosis were the most significant risk factors in this study. Only miliary tuberculosis was significantly associated with maternal mortality in pulmonary tuberculosis (p = 0.004, relative risk = 3.43) of 12 risk factors predicted to be associated with maternal mortality.

## Discussion

This study aimed to identify the risk factors associated with tuberculosis-related maternal mortality during pregnancy. The study found that patients with miliary tuberculosis had a 3.43-fold increased risk of death compared to patients without miliary tuberculosis. However, this study found that maternal age, gestational age, parity, and mode of delivery were not associated with pulmonary tuberculosis mortality in pregnant women. This study revealed that tuberculosis cases with HIV co-infection had a higher mortality rate than tuberculosis cases without HIV. Additionally, the HIV-positive group had more severe complications than the HIV-negative group. This study also found no significant difference between the survival and non-survival groups regarding the risk factors for heart disease during pregnancy. Moreover, the non-survivor group had a higher proportion of tuberculosis cases with pneumonia co-infection, although the difference was not significant.

This study found that age was not associated with pulmonary tuberculosis mortality in pregnant women and that there were no significant differences in gestational age and parity between the tuberculosis group that survived and the tuberculosis group that did not. Demographically, multipara dominated both groups. Nonetheless, 50% of non-surviving cases were reported at a younger gestational age (less than 28 weeks). Pregnancy may inhibit the pro-inflammatory T-helper1 cell response, which may mask tuberculosis symptoms while increasing susceptibility to new infections and tuberculosis reactivation [8]. Several alterations in cellular immunity occur during the final stage of pregnancy, including an increase in phagocyte number and activity, plasmoid dendritic cells, and a decrease in natural killer cell cytotoxicity and interferon-gamma production. This demonstrates the suppression of innate cellular immunity [9].

There was no significant difference between the survival and non-survival groups in terms of mode of delivery. Previous research found no significant difference between pregnant women with tuberculosis and pregnant women without tuberculosis in terms of mode of delivery. There were no significant postpartum complications, except for one case of postnatal maternal death due to shock and end-organ failure as a consequence of military tuberculosis [10]. According to a different study, tuberculosis was associated with the mode of delivery (vaginal delivery, lower segment cesarean section, and instrumental vaginal delivery), with a p-value of 0.009 [11].

Multiple variables influence the impact of tuberculosis on pregnancy, including disease severity, site of infection, human HIV co-infection, gestational age, initial therapy, and patient compliance [12]. This study revealed that tuberculosis cases with HIV co-infection had a higher mortality rate than tuberculosis cases without HIV. Additionally, the HIV-positive group had more severe complications than the HIV-negative group. According to a previous study conducted in Sudan, 11.9% of pregnant women with tuberculosis were also HIV positive. The rate of maternal mortality due to tuberculosis was 4.8%, and all of these deaths were due to co-infection with HIV [13]. Women with HIV and latent *Mycobacterium tuberculosis* infections are more likely to develop active tuberculosis during pregnancy [14]. The difference between our study and previous studies may be that HIV status information was not available for all samples. Thirteen pregnant women had unknown HIV status.

In this study, one of the 16 deceased patients had a history of mitral stenosis, pulmonary hypertension, and pneumonia. Pregnancy with heart disease has unfavorable outcomes, particularly in cases in which heart disease and tuberculosis infection worsen the mother’s condition. Pregnancy heart disease is a significant factor in maternal mortality after preeclampsia [15]. According to previous research, the prevalence of heart disease in pregnant women at Dr. Soetomo General Hospital is approximately 0.5%, with heart disease accounting for 14% of all maternal deaths [16].

In women with heart disease, pregnancy-related physiological changes may increase cardiac load and cause perinatal complications [17]. In the third trimester, increased cardiac load causes chronic maternal hypoxia, which is further exacerbated by tuberculosis. Compared with complications from tuberculosis alone, maternal mortality due to underlying heart disease may be a significant cause of death. However, this study found no significant difference between the survival and non-survival groups regarding the risk factors for heart disease during pregnancy. The limited number of heart disease cases may account for this finding.

The non-survivor group had a higher proportion of tuberculosis cases with pneumonia co-infection, although the difference was not statistically significant. Other factors may also contribute to pulmonary tuberculosis mortality in pregnant women. A 2016 retrospective study found that pneumonia was the primary predictor of mortality in patients with tuberculosis [6]. Patients with tuberculosis are more susceptible to bacterial infections, such as *Streptococcus pneumonia*, which can activate latent tuberculosis, and vice versa. Consequently, diagnosis and treatment are challenging [18]. Pneumonia, pulmonary edema, pleural effusion, tuberculosis, and asthma are frequent lung diseases that occur during pregnancy and are associated with poor maternal and fetal outcomes [19].

This study found that patients with miliary tuberculosis had a 3.43-fold increased risk of death compared to patients without miliary tuberculosis. According to previous studies, cavities, miliary tuberculosis, and pneumonia are predictors of tuberculosis-related mortality. Extrapulmonary involvement and cirrhosis of the liver also contribute to deaths caused by tuberculosis [6]. Another study reported a case of miliary tuberculosis with a negative outcome during pregnancy. In this case, ascites, epistaxis, melena, convulsions, and abnormal laboratory findings (hemoglobin 7% and thrombocyte 60,000/mm^3^) were reported, and maternal death occurred after delivery of a stillborn infant [20].

Age, immunodeficiency, diabetes, mental disorders, elevated liver enzyme levels, kidney dysfunction, malnutrition, thrombocytopenia, and radiological findings of ground-glass opacity are poor prognostic factors for miliary tuberculosis [21,22]. Multiorgan failure, septic shock, and acute respiratory distress syndrome are examples of fulminant acute conditions. With proper treatment, the mortality rate of miliary tuberculosis decreases from close to 100 percent to 7.1-30% [23].

Strengths and limitations

Indonesia is a high-burden country for tuberculosis and ranks among the countries with the highest incidence of the disease. Since the past decade, only a handful of studies have examined tuberculosis during pregnancy in Southeast Asia, and the majority of these studies are case reports or case series. This study had a larger sample of pregnant women with tuberculosis than the previous study on the same subject conducted in Indonesia or Southeast Asia. This cohort retrospective study found that miliary tuberculosis was significantly associated with maternal mortality in pulmonary tuberculosis (p = 0.004), indicating that patients with miliary tuberculosis have a 3.43-fold increased risk of death compared with patients without miliary tuberculosis. This study has some limitations. As a retrospective study, the data collected were limited. Additional prospective studies are required to further identify the risk factors associated with maternal mortality due to tuberculosis during pregnancy.

Research with larger samples should be conducted in the future to investigate why the incidence of miliary tuberculosis is higher in some regions of the world, such as Southeast Asia. Besides poor access to healthcare or inadequate treatment, there may be other factors that play a role in the development of miliary tuberculosis, such as genetics. Identifying the risk factors associated with tuberculosis-related maternal mortality during pregnancy can help improve the outcomes of pregnant women with tuberculosis.

## Conclusions

In summary, compared to patients without miliary tuberculosis, those with miliary tuberculosis have a 3.43-fold increased risk of death. The risk of developing severe complications from tuberculosis is especially high in developing countries; therefore, early detection of either the onset of the disease or the onset of complications is required to reduce the mortality rate. Unfortunately, pregnancy can further mask tuberculosis symptoms, making diagnosis difficult. Consequently, tuberculosis is frequently misdiagnosed and inadequately treated during pregnancy. If the patient receives appropriate treatment, it can reduce the mortality rate due to tuberculosis and its complications, such as miliary tuberculosis.
